# Prehospital factors associated with an ICU admission from the emergency department

**DOI:** 10.1186/cc14485

**Published:** 2015-03-16

**Authors:** TA Williams, J Finn, D Fatovich, D Brink, KM Ho, H Tohira

**Affiliations:** 1 Curtin University, Bentley, Australia; 2 Royal Perth Hospital, Perth, Australia; 3 St John Ambulance - WA, Belmont, Australia; 4 Curtin University Health Sciences, Bentley, Australia

## Introduction

This study aimed to describe the patient characteristics and prehospital factors associated with an ICU admission from the ED. There is a paucity of information about the early recognition of critical illness by paramedics; especially in the Australian prehospital setting.

## Methods

A retrospective cohort study, July 2012 to June 2014, conducted in the Perth metropolitan area, which is served by a single ambulance service. Adult patients were included if transported to a public hospital ED that used the ED information system (EDIS) (seven of eight EDs) and were admitted to the ICU from the ED (ED-ICU group). Patients aged <16 years, those from rural areas or transfers were excluded. We used existing ambulance clinical data linked to EDIS data. Prehospital cohort characteristics are described using univariate statistical techniques. Logistic regression was conducted with admission to the ICU from the ED (critical illness surrogate) as the outcome variable. Variables included in regression models were age, sex, paramedic-identified urgency, that is the time patients should be seen by a doctor based on the Australasian Triage Scale, paramedic-identified patient problem and the time taken from the ambulance service receiving the call to hospital arrival. Physiological variables: systolic blood pressure (SBP), heart rate (HR), respiratory rate (RR), temperature, oxygen saturation, and GCS were included in the logistic models.

## Results

Of the 142,448 eligible patients transported by ambulance, 1,076 (0.75%) were admitted to the ICU from the ED: the ED-ICU group was younger (mean 53 vs. 61 years, *P *< 0.001). Seventeen percent of ICU patients were transported as Urgency 1 (resuscitation/immediate) and 58% as Urgency 2 (within 10 minutes) while 70% of non-ICU patients were transported as Urgency 3 to 5 (*P *< 0.001). Thirteen percent of ICU patients had a SBP <90 mmHg, 15% had a HR ≥130 and 19% had a RR >30. Drug overdose (21%) and respiratory conditions (18%) were the most common ICU conditions identified by paramedics for the ED-ICU group. All variables entered into the logistic models were significant (all *P *< 0.001) except the time taken from receiving the call to hospital arrival (*P *= 0.48).

## Conclusion

Three-quarters of the ED-ICU patients were transported to the ED with high urgency. Currently no prehospital severity of illness or early warning system (EWS) is used in our ambulance service. Given the small proportion of ED-ICU patients who presented with abnormal observations, it is unlikely that introducing an EWS would alter practice or patient outcome.

